# Skull Base Approaches for Tuberculum Sellae Meningiomas: Institutional Experience in a Series of 34 Patients

**DOI:** 10.3390/life12040492

**Published:** 2022-03-28

**Authors:** Shamsul Alam, Gianluca Ferini, Nur Muhammad, Nazmin Ahmed, Abu Naim Mohammad Wakil, Kazi Mohammad Atiqul Islam, Mohammad Samsul Arifin, Abdullah Al Mahbub, Riad Habib, Mosiur Rahman Mojumder, Atul Vats, Bipin Chaurasia

**Affiliations:** 1 Department of Neurosurgery, Bangabandhu Sheikh Mujib Medical University, Dhaka 1000, Bangladesh; dr_shamsul@hotmail.com (S.A.); wakilan@yahoo.com (A.N.M.W.); rhkgs001@gmail.com (R.H.); 2 Department of Radiation Oncology, REM Radioterapia SRL, Viagrande, 95029 Catania, Italy; 3 Department of Neurosurgery, National Institute of Neurosciences and Hospital, Agargaon, Dhaka 1207, Bangladesh; nurmuhammadbsmmu@gmail.com (N.M.); kazidmc@gmail.com (K.M.A.I.); arifin62dmc@yahoo.com (M.S.A.); abdullahalmahbubbipash@gmail.com (A.A.M.); 4 Department of Neurosurgery, Ibrahim Cardiac Hospital and Research Institute (A Centre for Cardiovascular, Neuroscience and Organ Transplant Units), Shahbag, Dhaka 1000, Bangladesh; nazmin.bsmmu@gmail.com; 5 Department of Neurosurgery, Comilla Medical College Hospital, Comilla 3500, Bangladesh; mosiur.rahman.neurosurgeon@gmail.com; 6 Department of Neurosurgery, James Cook University Hospital, Middlesbrough TS4 3BW, UK; vatsatul7@gmail.com; 7 Department of Neurosurgery, Bhawani Hospital and Research Centre, Birgunj 44300, Nepal; trozexa@gmail.com

**Keywords:** tuberculum sellae, skull base, meningioma, transsphenoidal, endoscopy, microsurgery, pterional craniotomy, bifrontal craniotomy, supraciliary keyhole

## Abstract

(1) Background: The aim of the present study was to evaluate our institutional outcome in tuberculum sellae meningioma (TSM) patients treated microsurgically using multiple skull base approaches, including a transcranial approach and an extended endonasal transsphenoidal approach. (2) Materials and Methods: This is a retrospective study that includes 34 patients with TSM. The study aimed to observe the efficacy of the different common approaches used by a single neurosurgeon. All the patients were evaluated preoperatively and during follow-up with campimetry, head CT scan, and post-contrast MRI. (3) Results: After a transcranial approach, visual acuity improved in 86.20%, was stable in 10.34%, and deteriorated in 3.45%. Through transsphenoidal surgery, vision improved in 80%, was static in 20%, and deteriorated in 0%. Transcranial approaches included pterional, mini-bifrontal basal, and supraciliary keyhole microscopic craniotomies. Gross total removal was performed in 58.82%, near total in 10.34%, and partial removal in 3.45%. The transcranial/supraciliary keyhole endoscopic-assisted approach showed a gross total removal rate of 80%, and near total in 20%. The transsphenoidal approach showed a gross total removal rate of 60%, near total in 20%, and partial removal in 20%. (4) Conclusion: Endoscopic-assisted keyhole supraciliary mini craniotomy for resection of tuberculum sellae meningioma offers low morbidity and good visual outcome. The endonasal route is preferred for the removal of TSM when they are small and midline placed. The major limitation of this approach is a narrow surgical corridor and the restriction on midline-placed lesions. Gross total removal was better achieved with mini-bifrontal basal and pterional craniotomies.

## 1. Introduction

**Figure 1:**
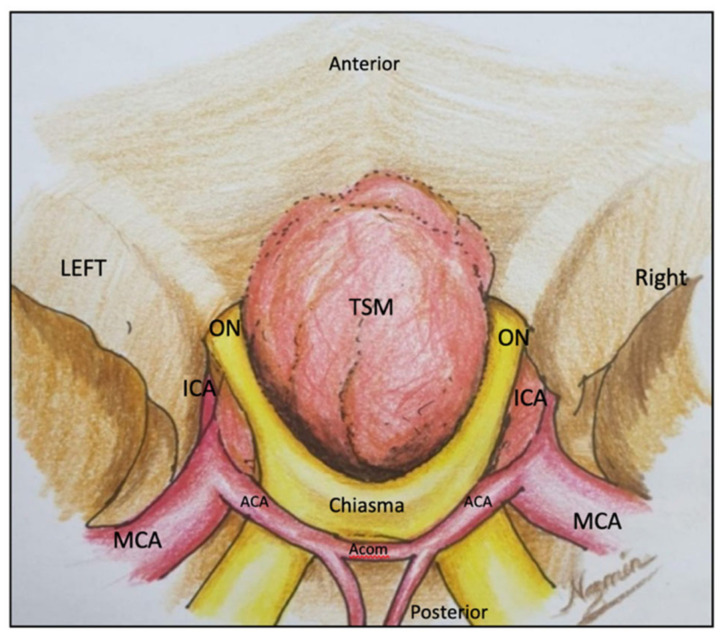
Depicted is a diagram of TSM compressing the optic chiasm backwards and downwards, internal carotid artery laterally, and anterior circulation complex upwards. In the figure: TSM—tuberculum sellae meningioma, ON—optic nerve, ICA—Internal carotid artery, ACA—anterior cerebral artery, MCA—middle cerebral artery, AComA—anterior communicating artery.

Tuberculum sellae meningiomas (TSM) account for 5–10% of all intracranial meningiomas and typically arise from the dura mater of tuberculum sellae, chiasmatic sulcus, and limbus sphenoidale [1,2,3,4,5]. Visual disturbance is the most common clinical presentation, up to 80% according to the series of Schick et al. [6], because of the intimate anatomical relation between tuberculum sellae and the optic apparatus. TSM in fact displace the optic apparatus upwards, and frequently up to 67% of cases invade the optic canals [7], leading to a decrease in visual acuity and visual field deficit [8] (Figure 1). The visual deficits are often asymmetric, reflecting the off-midline origin and then of the pattern of optic nerve and chiasmal compression [7]. Other less-common symptoms and signs are represented by headaches, dizziness, seizures, endocrine disturbances, altered behavior, and cranial nerve deficits [3,9,10,11,12,13]. The primary goal of surgery is to improve or at least maintain visual function, but this objective poses a formidable surgical challenge because of the risk of postoperative visual impairment [14]. Articles report that 10–20% of patients experience worsening of postoperative visual function [12]. Several authors have reported that unroofing of the optic canal and anterior clinoidectomies can improve visual outcome [15,16]. The aim of the present study was to evaluate the outcomes in TSM patients treated micro-surgically using multiple skull base approaches such as the transcranial approach and an extended endonasal transsphenoidal approach.

## 2. Materials and Methods

This is a retrospective study aimed to observe the efficacy of the different common approaches used by a single neurosurgeon. The approaches were a minipterional approach, a supraciliary keyhole microsurgical approach, an endoscope-assisted supraciliary keyhole approach, a bifrontal basal approach, and an extended endoscopic endonasal approach in resecting the tuberculum sellae meningioma. All the patients were evaluated preoperatively by campimetry and postcontrast MRI. Clinical postoperative follow-up included visual perimetry tests at one month and radiologically by postcontrast MRI at one and at six months. In some cases, due to the lack of economic resources, postcontrast CT head scans were performed.

### 2.1. Minipterional Approach

#### 2.1.1. Surgical Technique

The patient was positioned supine, with the head turned about 30° to the opposite side along the sphenoid wing and slightly (15°) retroflexed. The head was fixed in a three-pin Mayfield head holder. The scalp incision started just above the tragus, anterior to the superficial temporal artery, continuing superiorly behind the hairline and ending near to midline. Subgaleal and interfascial dissection was conducted to preserve the frontal branch of the facial nerve. The temporalis muscle was incised down to the periosteum, beneath the skin incision, and then the muscle and the pericranium were reflected anteriorly and inferiorly. The McCarty keyhole was used as a starting point for the craniotomy. The bone flap was cut and elevated. The dura mater was opened in a curved fashion with the sphenoid wing on its base. The next step was splitting the sylvian fissure to drain the cerebrospinal fluid. A representative case of our case series is presented below.

#### 2.1.2. Illustrative Case: 1

**Figure 2:**
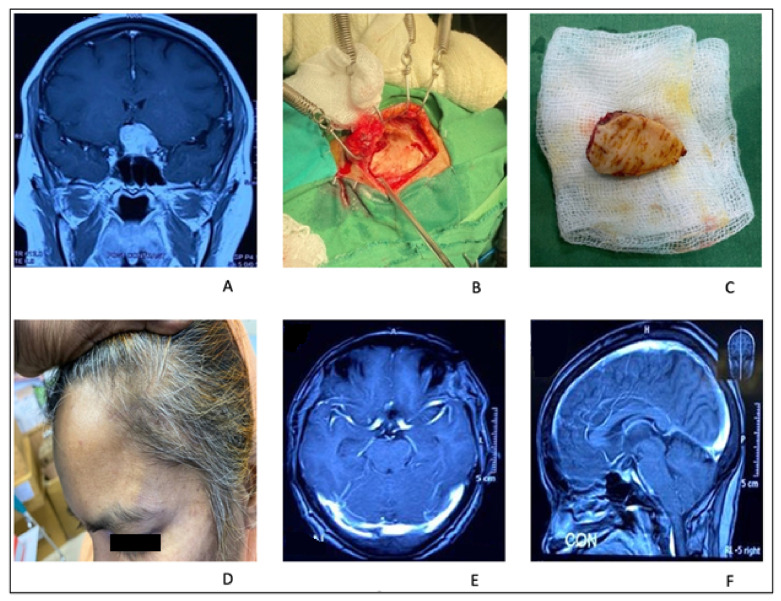
Coronal MRI image of a TSM showing extension of tumor through opticocarotid triangle on left side (**A**); Mini-pterional craniotomy on left side (**B**); Small size fronto-temporal craniotomy bone flap (**C**); Photograph of the patient showing healed scar (**D**); Post-operative axial and sagittal MRI with contrast showing no residual of the tumor (**E**,**F**).

A 55-year-old woman presented with gradual left eye blindness and occasional headaches. A brain MRI with Gadolinium revealed a TSM associated with left optic canal invasion. She underwent left sided minipterional craniotomy. Optic canal unroofing and anterior clinoidectomy were performed, and the tumor was devascularized from its attachment from the tuberculum sellae. The optic–carotid triangle area was exposed. The tumor was removed in piecemeal fashion, through the pre-chiasmatic and optic–carotid space. Gross total removal of the tumor was achieved. Her post-operative period was uneventful, and her vision improved to finger count (Figure 2).

### 2.2. Supraciliary Keyhole Microsurgical Approach

#### 2.2.1. Surgical Technique

The patient was placed in the supine position with the head turned 15–20° to the contralateral side and 15° retroflexed to allow the frontal lobe to slightly fall back. The head was fixed using the three-pin head holder. A curvilinear skin incision was made, and then one burr hole was placed at the McCarty’s point. The craniotomy was made in a standard fashion, of about 2 cm in length and 2 cm in height. If the frontal sinus was exposed intentionally or accidentally, the sinus mucosa was removed and cauterized, and the sinus was sealed with autologous and/or heterologous tissue. The dura was opened in a semilunar fashion with its base towards the orbital rim. The frontal lobe was slightly retracted to identify the suprachiasmatic cistern and opened to drain the CSF to give more brain relaxation. Arachnoid opening of the sylvian fissure started from medial to lateral. The opening of the sylvian fissure further facilitated the dissection and the anatomic exposure, revealing the major structures of the peritumoral area. The next step was to devascularize the tumor at its basal attachment. Tumor mass debulking was conducted with microsurgical technique.

#### 2.2.2. Illustrative Case 2

**Figure 3:**
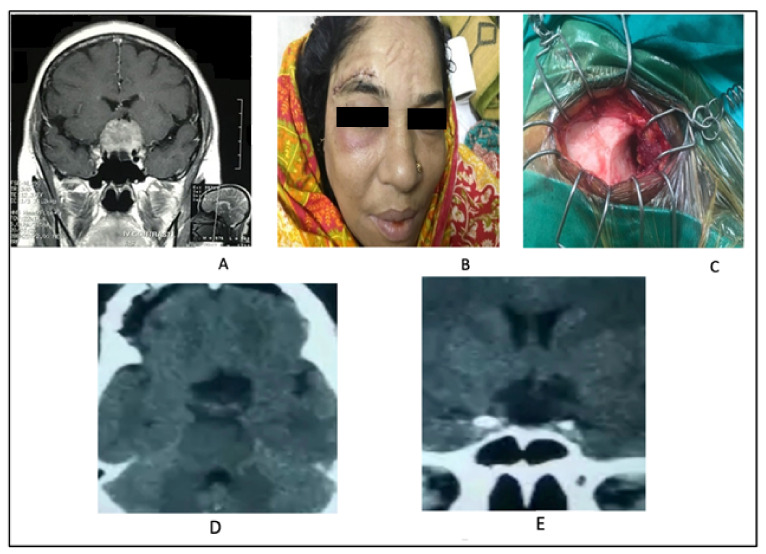
(**A**) Coronal view MRI with contrast showing a medium-sized TSM. (**B**) Showing a linear mark of supraciliary keyhole approach. (**C**) Per-operative picture of supraciliary keyhole exposure showing the frontal bone. (**D**) Post-operative CT scan of T.S meningioma showing no residual tumor in axial view. (**E**) No residual tumor in coronal view in post-operative CT scan.

A 45-year-old lady presented with a bitemporal field deficit along with mild headaches. Her brain MRI revealed the presence of a tuberculum sellae meningioma. She underwent keyhole supraciliary mini craniotomy and microsurgical removal of tuberculum sellae meningioma. Her postoperative recovery was excellent with visual outcome improvement (Figure 3).

### 2.3. Supraciliary Keyhole Endoscopic Approach

#### 2.3.1. Surgical Technique

This approach is like the supraciliary keyhole microsurgical approach, but here we used a 0° endoscope (4 mm wide, 18 cm length, Carl Storz, Tuttlingen, Germany). The endoscope was held by an endoscopic holder (Huidamed, Jintan, China), and the tumor dissection was carried out using micro instruments and micro scissors. Long bipolar forceps were used for cautery. The patient was placed in the supine position with the head turned 15–20° to the contralateral side and 15° retroflexed to allow the frontal lobe slightly to fall back. Patient position, supraorbital incision, and the size of craniotomy were like in the supraciliary keyhole microsurgical approach. Here, CSF was gently sucked out from the basal cistern to make frontal lobe slack, and the endoscopic micro instruments were manipulated in between the space of frontal bone and frontal lobe.

#### 2.3.2. Illustrative Case 3

**Figure 4:**
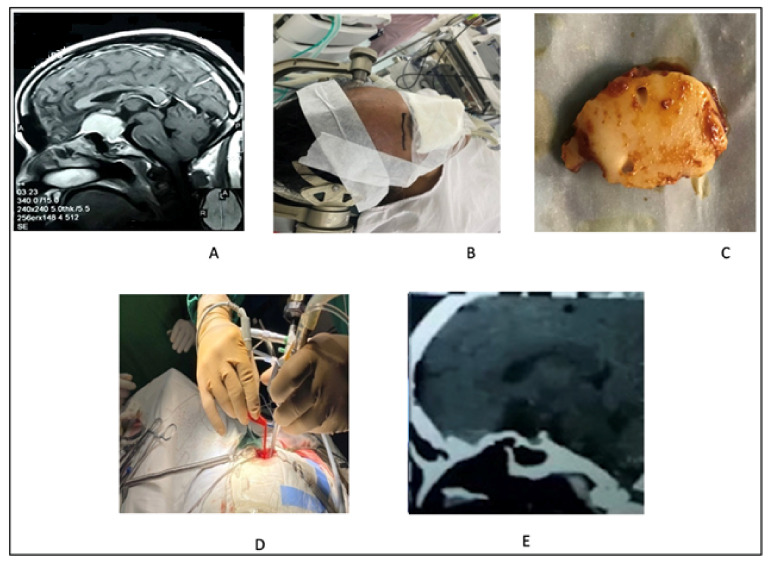
(**A**) Sagittal view MRI with contrast shows medium-sized TSM. (**B**) Planning for superciliary keyhole endoscopic approach. (**C**) Small piece of frontal bone in keyhole approach. (**D**) Picture shows three hand technique in superciliary keyhole endoscopic-assisted approach. (**E**) Post-operative CT scan in sagittal view showing no residual tumor.

A 35-year-old woman presented with a bitemporal field defect. Head MRI revealed TSM. She underwent an endoscopic-assisted supraciliary keyhole approach. The tumor was resected in piecemeal fashion. Her recovery was excellent and visual improvement was satisfactory (Figure 4).

### 2.4. Minibifrontal Basal Approach

#### 2.4.1. Surgical Technique

With the patient in a supine position, the head was fixed in a three-pin head holder. A midline osteotomy was performed, extending close to the orbital roof. Two burr holes were placed on the orbital bottoms on each side through a bicoronal skin incision beyond the hair line. The dura was opened parallel to the base and the sagittal sinus was ligated and cut at the cecal foramen, followed by transection of the falx, preserving the bridging veins. The frontal poles were retracted gently under magnification, using two self-retaining retractors adjusted stepwise. The interhemispheric and the bilateral olfactory cisterns were opened to drain the CSF. We investigated two cases using the bifrontal basal approach.

#### 2.4.2. Illustrative Case 4

**Figure 5:**
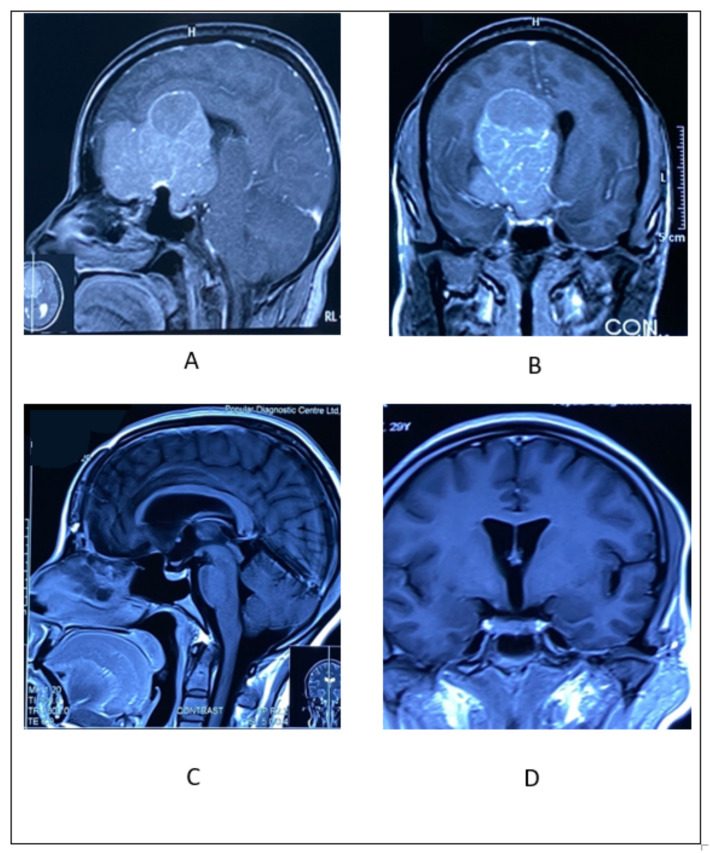
(**A**) Pre-operative sagittal view MRI with contrast showing huge TSM extending upward to the corpus callosum. (**B**) Pre-operative coronal view MRI with contrast showing huge TSM extending towards right frontal lobe. (**C**) Post-operative sagittal view showing no residual tumor and (**D**) Post-operative coronal image showing normal orientation of pituitary gland and hypothalamus.

A 56-year-old woman presented at our institution complaining about an impaired level of consciousness and a prolonged history of headaches and visual disturbances. Brain MRI revealed a huge tuberculum sellae meningioma with diffuse perilesional edema. She underwent an emergency minibifrontal craniotomy and tumor removal in piecemeal fashion. Her recovery was good, and her vision improved (Figure 5).

### 2.5. Extended Endoscopic Endonasal Approach

#### 2.5.1. Surgical Technique

A 0° endoscope, 4 mm in diameter (Carl Storz, Tuttlingen, Germany), was used freehand. Once the endoscope had been inserted into the chosen nostril, the inferior and middle turbinates, and the nasal septum were identified. Using Tilley forceps, long cotton pledgets, soaked in diluted adrenaline (1/10,000) or xylometazoline hydrochloride, were inserted in the space between the middle turbinate and the nasal septum to achieve a vasoconstrictive effect of these richly vascularized structures. The middle turbinate was gently medialized or removed to make sure that the surgical corridor that passes between the nasal septum and the turbinate itself was wide enough. Once the cotton pledgets were removed, adequate inspection of the posterior portion of the nasal cavity was conducted, and the choana, the sphenoethmoidal recess, and the sphenoid ostium were identified. After a careful inspection, a nasoseptal/Hadad flap was taken starting from the choana to the floor of the nasal septum, then turned upward near to the roof of nasal septum. The Hadad flap was parked into the nasopharynx.

The sellar floor was drilled and opened initially using a small diamond drill, and then enlarged with Kerrison bone punches. Once inside the sphenoid, the key anatomical landmarks were identified, including the planum sphenoidale, tuberculum sellae, sellae, lateral optico-carotid recesses (OCRs), optic canals, and clinoidal carotid protuberances. The bone from the mid-to-upper sellae was removed with a combination of the diamond drill and the Kerrison punches. Next, the bones of the tuberculum sellae and planum were removed. This is often hyperostotic and could be vascularized due to the adjacent tumor feeder from the ethmoidal arteries. The use of a diamond burr is safer than a cutting burr when working around critical neurovascular structures and is also helpful for bony hemostasis. The bone overlying the proximal optic canals would be unroofed to access the tumor extension into the medial optic canals bilaterally. Drilling with continuous irrigation should be performed to avoid thermal injury to the optic nerves. The lateral OCRs help estimate the location of the canalicular segment of the optic nerve. A key to achieving wide access to the optico-carotid cistern was to remove the lateral strut of the tuberculum sellae. The dura was then incised as cruciate or in a rectangular fashion. The tumor was removed in piecemeal fashion and bleeding was secured. Surgicel was used as a hemostatic agent 44. The gasket seal technique was used to close the dural defect, aided with fibrin glue. Merocel nasal pack was introduced as a mucosal hemostat.

#### 2.5.2. Illustrative Case 5

**Figure 6:**
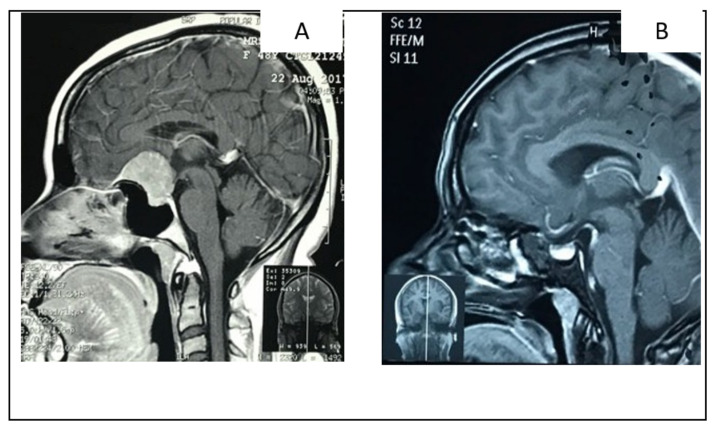
(**A**) Sagittal MRI with contrast in sagittal view showing small- to medium-size TSM, compressing of the pituitary gland. (**B**) Sagittal MRI with contrast in sagittal view showing no residual tumor following extended endonasal approach.

A 45-year-old lady was presented with bilateral visual impairment and occasional headaches. A head MRI revealed a small-size TSM. She underwent an extended endonasal approach and gross total removal of the tumor. Her vision improved significantly in the post-operative period (Figure 6).

## 3. Results

Data were collected from 2015 to 2020 and performed by a single operator. We retrieved 34 patients affected by TSM. Patient’s age distribution ranged from 11 to 65 years. Most of the patients were aged between 31 to 50 years (61.76%) (Table 1). Among them, 28 were females and 6 were males. Most of the patients were female, 82.35% (Table 2). Most patients presented with bitemporal visual field defects. Some were one-eye blind, and six cases (17.64%) were both-eyes blind (Table 3). The most frequently used approach was transcranial microsurgical (70.58%), followed by transsphenoidal (14.70%) and transcranial/supraciliary keyhole endoscopic-assisted (14.70%) (Table 4). Thirty-two tumors were small sized, while two were large sized (more than 6 cm) only. (Table 5). Vascular encasement was present in 32.35% and no vascular encasement was in 67.64% (Table 6). Vascular encasement was mainly in the form of arachnoid adhesion of the tumor with blood vessels. In a few cases there was circumferential adhesion of blood vessels to the tumor. After transcranial surgery, vision improved in 86.20%, was stable in 10.34%, and deteriorated in 03.45%. In the transsphenoidal surgery group, vision improved in 80%, was stable in 20%, and deteriorated in 0% (Table 7). In our series, through transcranial microsurgical approaches (pterional, mini-bifrontal basal, supraciliary keyhole microsurgical), GTR was conducted in 58.82%, near total in 10.34%, and partial removal in 3.45%. In the transcranial/supraciliary keyhole endoscopic-assisted approach groups, GTR was achieved in 80% and near total in 20%. The transsphenoidal approach group showed a GTR rate of 60%, near total in 20%, and partial removal in 20% (Table 8). The cause of partial or incomplete removal was due to tumor consistency, and in some cases difficulty in dissection in case of vascular encasement. Among the transcranial approaches, CSF leak presented in 6.89% of cases, while meningitis presented in 10.34%. In the transsphenoidal approach group, CSF leak was present in 20% of cases and meningitis in 20%. No mortality was present in both approaches at 30 days (Table 9).

## 4. Discussion

TSMs are relatively common and are a formidable surgical challenge [17,18,19,20,21,22]. As with most other cranial base lesions, TSMs have a relatively innocuous clinical presentation, despite their commonly encountered large size, due to the slow growth that characterizes the histology of these tumors. The neurological, visual, and long-term outcomes are determined by a combination of surgical techniques, tumor consistency, and relation/encasement with the surrounding neuro-vascular structures. The extent of the surgical resection of the tumor may affect the tumor recurrence and regrowth. However, new radio-surgical techniques offer the possibility to improve tumor control; thus, a radical resection should not be pursued in all cases, rather tailored in a patient–tumor-specific fashion [23,24]. A preponderance of TSM in women has been uniformly observed in previous reports [5,25,26,27], and further confirmed in our series (82.35%). As described in previous series, most of these tumors were encountered in patients in the third to fifth decades of life [25,26,28]. In our study, most of the patients were aged between 31 to 50 years (61.76%).

### 4.1. Minipterional Approach: Indications

We prefer this approach when the TSM has significant extension towards the optic canal and the optic–carotid recess to the parasellar area. In this case, we need to accomplish an optic foramen decompression to remove the meningioma from the optic canal and remove the tumor lateral to the optic nerve and carotid artery. A minipterional craniotomy and a transylvian approach provide a favorable corridor to deal with optic foramen and the optic–carotid recess area.

### 4.2. Supraciliary Keyhole Microsurgical Approach: Indications

This technique is a medial approach to find the tuberculum sellae meningioma. We choose this approach for small meningiomas, midline located, and with no or minimum ICA involvement or displacement.

### 4.3. Supraciliary Keyhole Endoscopic Approach: Indications

We prefer this technique when the TSM is extended to the sellae and mainly occupies the sellae, while directing posteriorly with no or less A-Com complex encasement. It gives us direct exposure to the sellae and tuberculum sellae. We use a three-hands approach where a 0-degree endoscope is held by an endoscopic holder or by an assisting surgeon. The operator works bimanually with the endoscope and dedicated tools.

### 4.4. Minibifrontal Basal Approach: Indications

We choose this approach when the TSM is greater than 4 cm, with perilesional edema and partial or total obliteration of the frontal horn of the lateral ventricle. The neck is needed to be hyperextended to achieve better exposure of the tumor.

### 4.5. Extended Endoscopic Endonasal Approach: Indications

We prefer this approach when the TSM is extended to the sellae, occupying the sellar region, directing posteriorly towards the third ventricle with no or minor A-Com complex encasement. We perform a three-hand approach where a 0-degree endoscope is held by an assistant surgeon and the operator works bimanually.

Younger patients tolerated the surgical procedure better than older patients, in which a more conservative resection associated to complementary radiosurgery should be considered in the surgical planning. Systemic factors, such as hypertension and diabetes mellitus, could affect the surgical procedure and outcome to a certain degree. The extent of visual deficit was the single most important factor that determined the course of surgery. The entity of the involvement of the optic nerve and of the associated visual deficit is strongly related with a tighter relationship of the neurovascular structures (optic nerve, the internal carotid artery, and its branches) with the tumor, and consequently with a more difficult dissection. Visual symptoms arise early and usually are slowly progressive [29,30,31,32,33]. However, because of the absence or subtleness of other symptoms, these tumors can remain undiagnosed for a significant period. It was seen that in patients with a longer duration of visual symptoms, the tumors were relatively firm and the relationship with adjoining structures was more intense. Various authors have suggested the size of the tumor to be a reliable predictive factor for surgical difficulties [18,20,22,25,34,35]. Large tumors cause more severe stretching of the adjoining nerves and vessels, and consequently, the resections were more difficult in our series. Al-Mefty and Smith’s series (1999) showed a 91% resection rate, 25% visual improvement, and 8.6% mortality [35]. Mathiesen and Kihlström’s series (2006) revealed a 90% resection rate, 75.9% visual improvement and 0% mortality 28 (Table 10). In agreement with the previous literature, in our series visual recovery was better in patients whose preoperative vision was relatively good [5,36,37]. In most of the cases, the pituitary stalk was separated from the tumor with a well-defined arachnoid plane and was never encased by the tumor. Meta-analysis of different series by de Divitiis et al. revealed that visual improvement was 58.4% and worsening in 12.9%, with a lesion removal rate of 87.6%, rare CSF leaks, and a mortality of 2.7% in transcranial group. Transsphenoidal approaches had a visual improvement of 75% and worsening in 0%, a lesion removal rate of 93.1%, a CSF leak of 20%, and mortality of 3% [38]. Compared to the literature reports, the present series show a better visual improvement rate, a lower postoperative vision worsening, and a similar CSF leak in transsphenoidal surgery. Mortality in our series is nil.

## 5. Conclusions

Endoscopic-assisted keyhole supraciliary minicraniotomy for resection of tuberculum sellae meningioma offers low morbidity and good visual outcome. The endonasal route is preferred for the removal of TSM when they are small and midline placed. The major limitation of this approach is a narrow surgical corridor and the restriction to midline-placed lesions. Gross total removal was better achieved with minibifrontal basal and pterional craniotomies. The arachnoid surrounding the optic nerves, chiasm, and anterior circulation artery must be spared to improve the visual function postoperatively.

## Figures and Tables

**Table 1:** Age distribution (N—34).

No of Patients	
Age Group(years)	Frequency
11–20	1
21–30	6
31–40	11
41–50	10
51–60	5
61–70	1
Total	34

**Table 2:** Distribution of patients by sex (N—34).

No of Patients		
Sex	Frequency	Percentage (%)
Male	6	17.64
Female	28	82.35
Total	34	100
Male:Female	1:4.66	

**Table 3:** Distribution of visual presentation.

Visual Presentation	No of Patients
	Frequency (Percentage %)
No visual field defect	4 (11.76%)
Bilateral blind	6 (17.64%)
Bitemporal field defect	16 (47.05%)
Tunnel vision	8 (23.52%)
Total:	34 (100%)

**Table 4:** Distribution of operative approaches (N—34).

Transcranial Approach (%)	Transsphenoidal Approach. (%)
Microscopic	(70.58%)
Endoscopic-assisted	(14.70%)
transcranial/supraciliary keyhole endoscopic-assisted	(14.70%)
Total:	(85.29%)

**Table 5:** Distribution of tumor size (N—34).

No of Patients		
Size	Frequency	Percentage (%)
<3 cm	15	(44.11%)
3–6 cm	17	(50%)
>6 cm	2	(5.88%)
Total:	34	100%

**Table 6:** Distribution of vascular encasement (N—34).

No of Patients (%)		
	Yes	No
Vascular encasement	11 (32.35%)	23 (67.64%)

**Table 7:** Distribution of visual outcome (N—34).

No of Patients (%)		
	Transcranial	Transsphenoidal
Improved Vision	25 (86.20%)	4 (80%)
Static vision	3 (10.34%)	1 (20%)
Deteriorated vision	1 (03.45%)	0 (%)

**Table 8:** Distribution of extent of tumor removal (N—34).

No of Patients (%)			
	Transcranial Microscopic	Transcranial/Superciliary Keyhole Endoscopic-Assisted	Transsphenoidal
Gross total	20 (58.82%)	4 (80%)	3 (60%)
Near total	3 (10.34%)	1 (20%)	1 (20%)
Partial	1 (03.45%)	0	1 (20%)

**Table 9:** Distribution of complication (N—34).

No of Patients (%)		
	Transcranial	Transsphenoidal
CSF leak	2 (6.89%)	1 (20%)
Meningitis	3 (10.34%)	1 (20%)
Vascular injury	Nil	Nil
Would infection	2 (6.89%)	Nil

**Table 10:** Summary literature on multiple skull base approaches for TSM.

Series (Ref. No)	No of Cases	Approach	Gross Total Removal	Visual Outcome Improved	Complication Mortality
Al-Mefty and Smith, 1991 [39]	35	Transcranial	91%	25%	8.6%
Mathiesen and Kihlstrom, 2006 [40]	29	Transcranial	90%	75.9%	0%
Jho, 2001 [41]	1	Endoscopic endonasal transphenoidal	100%	100%	0%
Dusick et al., 2005 [42]	7	Microsurgical endoscopic-assisted	57.14%	Not recorded	0%
de Divitiis et al., 2007 [43]	44	Transcranial	86.4%	61.4%	0%
	11	Endoscopic endonasal	83%	71.4%	0%
Palani et al., 2012 [44]	41	Transcranial	73%	27%	4.9%
Our Series 2021	24	Transcranial microscopic	58.82%	86.20%	0%
	5	Transcranial endoscopic	80%	86.20%	0%
	5	Extended endonasal transsphenoidal approach	60%	80%	0%

## Data Availability

Data of this study would be available from corresponding author on reasonable request.
